# Long-Term Intraocular Pressure Changes Following Phacoemulsification in Eyes with and Without Primary Open-Angle Glaucoma

**DOI:** 10.3390/medicina62081525

**Published:** 2026-08-08

**Authors:** Sava Barišić, Sofija Davidović Terzić, Nikola Babić, Aleksandar Miljković, Vladimir Čanadanović

**Affiliations:** 1 Eye Clinic, University Clinical Centre of Vojvodina, 21000 Novi Sad, Serbia; sofija.davidovic@mf.uns.ac.rs (S.D.T.); nikola.babic@mf.uns.ac.rs (N.B.); aleksandar.miljkovic@mf.uns.ac.rs (A.M.); vladimir.canadanovic@mf.uns.ac.rs (V.Č.); 2 Faculty of Medicine, University of Novi Sad, 21000 Novi Sad, Serbia

**Keywords:** phacoemulsification, intraocular pressure, primary open-angle glaucoma, cataract surgery, prospective–retrospective cohort study, linear mixed-effects model, long-term follow-up

## Abstract

*Background and Objectives*: The effect of phacoemulsification on long-term intraocular pressure (IOP) in primary open-angle glaucoma (POAG) remains incompletely defined, particularly in medically controlled disease. This study aimed to evaluate long-term IOP changes following phacoemulsification in eyes with and without POAG using longitudinal statistical modelling. *Materials and Methods*: This single-centre study, comprising prospective and retrospective components, included 61 patients (30 controls and 31 with medically controlled POAG) who underwent uncomplicated phacoemulsification with intraocular lens implantation. Patients were followed prospectively through six months; long-term IOP values were obtained retrospectively from routine clinical follow-up records. IOP was measured preoperatively and at 1 week, 1 month, 3 months, 6 months, and at final follow-up (44–77 months). Longitudinal IOP changes were analysed using linear mixed-effects models with a piecewise time structure to account for repeated measurements and uneven follow-up. Predictors of postoperative IOP change were evaluated using multivariable linear regression. Changes in antiglaucoma medication burden were assessed using the Wilcoxon signed-rank test. *Results*: A significant overall effect of time on IOP was observed (*p* < 0.001), with a significant early postoperative reduction (*p* < 0.001) followed by long-term stabilisation. No time-by-group interaction reached statistical significance, indicating broadly similar longitudinal IOP trajectories in glaucomatous and non-glaucomatous eyes. Mean preoperative IOP was 14.80 ± 2.50 mmHg in controls and 15.48 ± 3.12 mmHg in the POAG group. At final follow-up (44–77 months), mean IOP was reduced by 1.20 mmHg (8.1%) in the control group and by 1.13 mmHg (7.3%) in the POAG group relative to preoperative values. At 60 months, model-predicted IOP remained below baseline in both groups (control: 13.55 mmHg; POAG: 14.39 mmHg). Higher preoperative IOP showed the strongest association with greater postoperative IOP reduction (*p* < 0.001). No significant long-term change in antiglaucoma medication burden was observed in the POAG group (*p* = 0.157). *Conclusions*: Uncomplicated phacoemulsification produced a modest but sustained reduction in IOP over long-term follow-up in eyes with and without medically controlled POAG. In POAG, IOP remained below baseline over long-term follow-up, with 29 of 31 patients requiring no change in the number of topical agents. Cataract surgery alone provides a limited but durable IOP benefit in medically controlled POAG.

## 1. Introduction

Cataract and glaucoma are chronic, progressive ocular diseases and remain among the leading causes of visual impairment worldwide [1]. Both conditions predominantly affect older individuals and frequently coexist within the same patient population [2,3,4]. While cataract is treated exclusively by surgery, glaucoma management is primarily based on lowering intraocular pressure (IOP) through medical and surgical interventions. Because IOP remains the only modifiable risk factor for glaucoma progression, understanding the effect of cataract surgery on postoperative IOP is of clinical relevance.

The influence of cataract extraction on postoperative IOP has been investigated for several decades [5,6]. Most studies describe a characteristic postoperative IOP course after uncomplicated phacoemulsification, with a possible early transient increase followed by a modest reduction below baseline, of variable duration [7,8,9]. In contrast, intraoperative or postoperative complications may lead to significant and sometimes permanent elevations of IOP [10,11]. These observations highlight the importance of both early and long-term postoperative IOP assessment, particularly in eyes with pre-existing glaucoma.

A systematic review by the American Academy of Ophthalmology summarised the longer-term IOP effect of phacoemulsification in treated glaucoma [12], while subsequent registry analyses indicate that early postoperative IOP spikes affect a minority of patients: in an analysis of 1,191,034 eyes, a spike occurred in 3.7% of all eyes and 5.2% of eyes with glaucoma [13]. Glaucoma and higher baseline pressure are consistent risk factors for such spikes [13], and postoperative pressure elevation has subsequently been associated with an increased risk of later open-angle glaucoma [14]. How much pressure ultimately falls, and for how long, varies considerably between individuals and across glaucoma subtypes.

In primary angle-closure glaucoma, phacoemulsification has been consistently associated with clinically meaningful IOP lowering [15,16,17,18]. In pseudoexfoliative glaucoma, substantial pressure reductions have also been reported, although the available series combined cataract surgery with trabecular aspiration or washout of exfoliative material rather than phacoemulsification alone [19,20]. In contrast, postoperative IOP changes in primary open-angle glaucoma (POAG) are less consistent, with most studies reporting relatively small reductions and substantial inter-individual variability [21,22,23,24,25,26]. A Bayesian analysis further emphasised the limited magnitude of the POAG effect by showing only a 0.1–3% probability of achieving a ≥5 mmHg reduction after phacoemulsification alone [27]. Consistent with these findings, long-term cohort data from normotensive and ocular hypertensive eyes have also shown only modest and variable reductions in IOP after phacoemulsification [28].

Several preoperative factors, including baseline IOP and anterior segment characteristics, have been proposed as predictors of postoperative IOP change [18,29]. However, the mechanism underlying postoperative pressure reduction, most probably improvement in trabecular outflow facility following lens removal, remains only partially characterised [30,31]. Moreover, inconsistencies in study design, follow-up duration, and statistical methodology, particularly inadequate handling of repeated measurements and irregular follow-up intervals, limit the interpretation and comparability of existing evidence.

Therefore, the present study aimed to evaluate long-term IOP changes following phacoemulsification with intraocular lens (IOL) implantation in patients with and without medically controlled primary open-angle glaucoma, using longitudinal statistical methods that account for repeated measurements and uneven follow-up, and to explore potential clinical and biometric predictors of postoperative IOP change.

## 2. Materials and Methods

The present study was a single-centre, comparative cohort study with both prospective and retrospective components, conducted at the Eye Clinic of the University Clinical Centre of Vojvodina in Novi Sad, Serbia. Consecutive patients undergoing cataract surgery between 2012 and 2015 were enrolled prospectively. Initially, 100 patients were included, comprising 50 controls and 50 patients with primary open-angle glaucoma, assigned according to glaucoma status. The prospective phase comprised standardised preoperative assessment and postoperative follow-up through six months after surgery. Several years after the initial study, long-term outcome data were obtained retrospectively from the patients’ routine clinical follow-up records. Among the original cohort, 61 patients (30 controls and 31 with primary open-angle glaucoma) had long-term follow-up data available and were included in the final analysis. The remaining 39 patients were unavailable for long-term evaluation owing to loss to follow-up or various personal or logistical reasons. These 61 patients constituted the final study cohort for longitudinal modelling of IOP changes. The variable length of long-term follow-up (44–77 months) reflects the retrospective use of routinely scheduled clinical visits rather than fixed research time points. The prospective phase was conducted as part of a doctoral research project [32] under separate ethics approval, while the subsequent retrospective long-term follow-up received its own dedicated ethics approval (see the Institutional Review Board Statement).

To assess potential attrition bias, baseline demographic and ocular characteristics of the 61 retained patients were compared with those of the 39 patients unavailable for long-term follow-up. Continuous variables were compared using Welch’s *t*-test or the Mann–Whitney U test, as appropriate based on normality testing (Shapiro–Wilk); categorical variables were compared using Fisher’s exact test. This comparison encompassed all preoperative parameters and IOP at the early postoperative time points (week 1, month 1, month 3, and month 6). All 61 analysed patients contributed six IOP measurements—one preoperative and five postoperative—so the analysed dataset was complete and no imputation was required. The linear mixed-effects framework was selected to account for within-patient correlation among repeated measurements and for the patient-specific timing of the final follow-up visit. Exploratory analyses were considered hypothesis-generating and were not adjusted for multiple comparisons. Sample size and statistical power are described below.

The control group consisted of patients with clinically significant cataract and no history of glaucoma or other ocular or systemic disease that could influence IOP. The glaucoma group included patients with clinically significant cataract and medically controlled primary open-angle glaucoma. Medically controlled glaucoma was defined as stable antiglaucoma therapy without changes and maintenance of target IOP during the two months preceding surgery. The diagnosis of primary open-angle glaucoma was based on a documented history of untreated IOP exceeding 21 mmHg, characteristic glaucomatous changes in the optic nerve head and retinal nerve fibre layer, an open anterior chamber angle with Shaffer grade ≥3 in all four quadrants on gonioscopy, and early to moderate visual field defects, staged by mean deviation according to the Hodapp–Parrish–Anderson thresholds (mean deviation better than −12 dB; defects worse than this threshold were classified as severe and excluded) [33,34]. Only one eye per patient was included in the study. When both eyes met the inclusion criteria, the eye with the more advanced, visually significant cataract—that is, the eye for which surgery was first indicated—was included; when cataract severity was comparable between the two eyes, the right eye was selected by convention. The indication for cataract surgery in all cases was visual acuity impairment due to lens opacification.

All surgical procedures were performed in a standardised manner by a single experienced surgeon (V.Č.). Phacoemulsification was carried out under retrobulbar block anaesthesia with 2% lidocaine and adrenaline (Lidokain 2%–Adrenalin; Galenika a.d., Belgrade, Serbia) using a 2.75 mm superior clear corneal incision and two side-port paracenteses created with a 15-degree tapered blade. A dispersive ophthalmic viscosurgical device (Viscoat, sodium chondroitin sulfate 4%–sodium hyaluronate 3%; Alcon Laboratories, Inc., Fort Worth, TX, USA) was used during the divide-and-conquer phacoemulsification technique. In all cases, a hydrophobic acrylic intraocular lens (AcrySof SA60AT; Alcon Laboratories, Inc., Fort Worth, TX, USA) was implanted in the capsular bag, followed by thorough aspiration of the viscoelastic material and intracameral administration of cefuroxime 1 mg in 0.1 mL (Nilacef, 1500 mg powder for solution for injection; Hemofarm A.D., Vršac, Serbia), prepared in the hospital pharmacy. Postoperatively, all patients received combined antibiotic–corticosteroid eye drops (TobraDex, tobramycin 0.3%–dexamethasone 0.1%; Alcon Laboratories, Inc., Fort Worth, TX, USA) eight times daily for one month, without tapering. Patients with primary open-angle glaucoma continued their preoperative antiglaucoma therapy without modification from the first postoperative day. No intraoperative or postoperative complications affecting IOP were recorded.

IOP was measured prospectively, under a standardised protocol, preoperatively and postoperatively at 1 week, 1 month, 3 months, and 6 months. All readings, at every time point, were obtained by Goldmann applanation tonometry using the same tonometer and at the same time of day (±2 h) to minimise diurnal and instrument-related variability. For the long-term visits, which were identified retrospectively from routine clinical follow-up, the tonometer used and the appointment time were verified against the clinical records for every included patient. Ocular biometric parameters—axial length, anterior chamber depth, and lens thickness—were obtained by contact ultrasound A-scan biometry (Sonomed A-Scan A2500; Sonomed Escalon, Lake Success, NY, USA) under topical anaesthesia, with keratometry recorded separately; each biometric value was taken as the average of repeated scans acquired along the visual axis. Long-term IOP, recorded at the final follow-up visit (44 to 77 months postoperatively), was obtained retrospectively from the patients’ routine clinical records; because these visits were not scheduled at fixed research intervals, the long-term follow-up duration varied between patients. The primary outcome of the study was longitudinal change in IOP following phacoemulsification. Secondary analyses evaluated potential predictors of postoperative IOP change, including preoperative IOP, preoperative and 1-month postoperatively measured anterior chamber depth, axial length, lens thickness, keratometry, corneal astigmatism, age, intraocular lens power, and best-corrected visual acuity assessed preoperatively and at 1 month postoperatively.

Antiglaucoma medication use was recorded preoperatively and at the final follow-up visit. Medication burden was quantified as the number of topical antiglaucoma agents used; each pharmacologically active ingredient was counted as one agent, so fixed-combination preparations were counted according to their number of active ingredients. Changes in medication burden over time in the primary open-angle glaucoma group were summarised descriptively (numbers of patients with an increase, no change, or a decrease) and additionally tested using the Wilcoxon signed-rank test (zero differences excluded, normal approximation). Because only two patients had a non-zero change, this test is approximate and is reported for completeness alongside the descriptive counts.

Continuous variables are presented as mean ± standard deviation and categorical variables as counts and percentages. Baseline between-group differences (Table 1) were assessed using Welch’s *t*-test or the Mann–Whitney U test for continuous variables, as appropriate based on normality testing (Shapiro–Wilk), and Fisher’s exact test for categorical variables. Longitudinal IOP measurements were analysed using linear mixed-effects models with a random intercept for each subject to account for repeated measures and unequal follow-up durations. Models were estimated by restricted maximum likelihood; random slopes were not specified, given the limited number of observations per subject. Time was treated as a continuous variable and modelled using a segmented (piecewise linear) approach with knots at 1 week and 6 months to estimate early, intermediate, and long-term postoperative IOP trends. Fixed effects included time, study group, and their interaction terms. The model intercept represents the estimated baseline IOP for the reference group. Predictors of long-term IOP change (final follow-up minus preoperative IOP) were evaluated using multivariable linear regression models incorporating relevant demographic and baseline ocular variables. A *p*-value < 0.05 was considered statistically significant. Analyses were performed in Python 3.12.3(Python Software Foundation, Wilmington, DE, USA; https://www.python.org, accessed on 1 August 2026) using the pandas version 3.0.2 (https://pandas.pydata.org, accessed on 1 August 2026), SciPy version 1.17.1 (https://scipy.org, accessed on 1 August 2026), and statsmodels version 0.14.6 (https://www.statsmodels.org, accessed on 1 August 2026) libraries. Artificial intelligence tools were used to assist with the literature search (Consensus, web version; Consensus NLP, Inc., Boston, MA, USA; https://consensus.app, accessed on 1 August 2026), generation of Python statistical code (Claude Sonnet 4.6; Anthropic, PBC, San Francisco, CA, USA; https://claude.ai, accessed on 1 August 2026), and language and grammar review (Grammarly, web version; Grammarly, Inc., San Francisco, CA, USA; https://www.grammarly.com, accessed on 1 August 2026). 

The a priori sample size was based on the study’s original design cohort of 100 patients (50 control and 50 POAG eyes). For the primary outcome—the within-group change in IOP after phacoemulsification—detecting a 1.5 mmHg change (standard deviation 1.8 mmHg) with two-sided α = 0.05 and 80% power required about 14 eyes per group, which the design cohort exceeded. In the present long-term sample of 61 eyes (30 control, 31 POAG), the achieved power for the observed IOP reductions was 0.78 (control) and 0.86 (POAG). The between-group difference in IOP change was very small (≈0.07 mmHg; Cohen’s d ≈ 0.03); with approximately 30 patients per group, the study was powered to detect only relatively large between-group differences (of the order of 1.6 mmHg); smaller differences cannot be excluded. The study was not designed as an equivalence or non-inferiority trial, and the precision of the group comparison is best judged from the confidence intervals reported in the Results.

## 3. Results

A total of 61 patients were included in the final analysis, comprising 30 patients in the control group and 31 patients with medically controlled primary open-angle glaucoma. Baseline demographic and ocular characteristics are summarised in Table 1. The two groups were comparable with respect to age, sex distribution, axial length, lens thickness, keratometry values, corneal astigmatism, intraocular lens power, and best-corrected visual acuity (all *p* > 0.05). Preoperative IOP was higher in the POAG group (15.48 ± 3.12 mmHg) than in controls (14.80 ± 2.50 mmHg); however, this difference did not reach statistical significance (*p* = 0.35). Preoperative anterior chamber depth was significantly shallower in eyes with POAG compared with controls (3.02 ± 0.28 mm vs. 3.23 ± 0.32 mm; *p* = 0.01). The mean number of antiglaucoma medications in the POAG group at baseline was 1.48 ± 0.68.

Comparison of the 61 retained patients with the 39 patients unavailable for long-term follow-up revealed no significant differences in sex, age, keratometry values, corneal astigmatism, best-corrected visual acuity, intraocular lens power, preoperative IOP, or the proportion of patients receiving antiglaucoma therapy (all *p* > 0.05). Retained patients had marginally longer axial length (23.49 ± 1.27 mm vs. 22.94 ± 0.88 mm; *p* = 0.026), with correspondingly lower IOL power (21.37 ± 2.20 D vs. 22.45 ± 2.34 D; *p* = 0.033), reflecting the expected inverse relationship between these parameters rather than an independent biometric difference. Preoperative anterior chamber depth was modestly greater in the retained group overall (3.12 ± 0.32 mm vs. 2.96 ± 0.28 mm; *p* = 0.007), driven primarily by a significant difference in the control subgroup (3.23 ± 0.32 mm vs. 2.90 ± 0.25 mm; *p* < 0.001); no such difference was found in the POAG subgroup (3.02 ± 0.28 mm vs. 3.02 ± 0.31 mm; *p* = 0.928). Critically, IOP did not differ significantly between retained and attrited patients at any time point assessed—preoperatively or at week 1, month 1, month 3, or month 6—either in the overall cohort or within either subgroup separately (all *p* > 0.05). These findings provided no evidence of differential attrition with respect to the measured IOP outcomes, although residual selection bias cannot be excluded.

**Figure 1:**
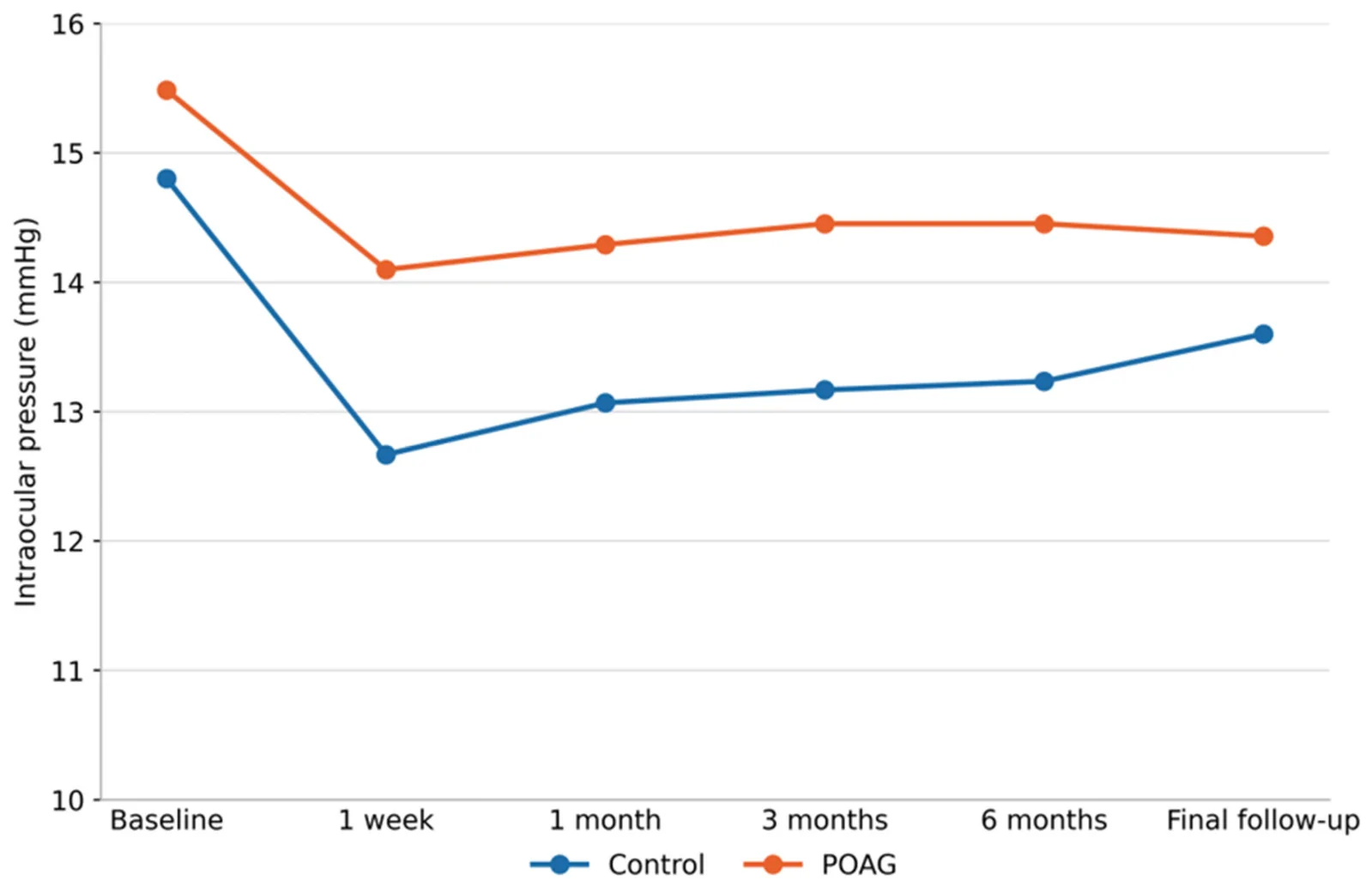
Observed longitudinal IOP values following phacoemulsification in control eyes and eyes with primary open-angle glaucoma.

Longitudinal analysis using a linear mixed-effects model demonstrated a significant overall effect of time on IOP following phacoemulsification (joint Wald test of all six time and time-by-group terms: χ^2^ = 88.3, 6 df, *p* < 0.001; the corresponding within-group tests gave χ^2^ = 60.6, 3 df, *p* < 0.001 in controls and χ^2^ = 27.7, 3 df, *p* < 0.001 in POAG eyes), reflecting a postoperative reduction in IOP. Fixed-effect estimates from the mixed-effects model are presented in Table 2. The model was fit to 366 IOP measurements from 61 eyes; a random intercept accounted for the substantial clustering of repeated measurements within eyes (intraclass correlation 0.76). The segmented time specification identified a steep, significant postoperative decline in IOP over the first postoperative week (slope: −7.82 mmHg/month; 95% confidence interval (CI): −9.83 to −5.81; *p* < 0.001), whereas no significant changes were observed during the intermediate (slope: +0.09 mmHg/month; *p* = 0.06) or long-term phases (slope: +0.004 mmHg/month; *p* = 0.49), indicating stabilisation over time. The time-by-group interaction was clearly non-significant during the intermediate and long-term phases (*p* = 0.55 and *p* = 0.47, respectively), indicating parallel IOP trajectories in glaucomatous and non-glaucomatous eyes over the bulk of follow-up. During the first postoperative week, a non-significant trend toward a smaller early pressure reduction was observed in POAG eyes (interaction coefficient: +2.70 mmHg/month; *p* = 0.061), corresponding to an early slope of −5.12 mmHg/month in glaucomatous versus −7.82 mmHg/month in control eyes. As this borderline difference did not reach statistical significance, the postoperative pressure courses were considered broadly comparable between groups. Observed mean IOP values at each follow-up visit are illustrated in Figure 1. Both groups exhibited a similar postoperative pattern, characterised by an early reduction in IOP followed by partial recovery and long-term stability. Absolute IOP values remained consistently higher in the POAG group throughout the follow-up period; however, the magnitude and temporal pattern of postoperative change were similar between groups. Despite intensive topical corticosteroid treatment (eight times daily, without tapering) throughout the first postoperative month, no IOP elevation was observed at the scheduled one-week or one-month visits in either group; mean IOP was already reduced at the one-month visit relative to baseline. Transient pressure elevations between scheduled visits cannot be excluded.

Model-based predicted IOP values at selected postoperative time points are shown in Table 3. These estimates confirmed a sustained reduction in IOP relative to baseline through long-term follow-up in both groups. At the one-week visit—the lowest scheduled postoperative mean—model-predicted IOP was 12.84 mmHg (95% CI: 12.06–13.62) in controls and 14.20 mmHg (95% CI: 13.43–14.97) in the POAG group. At the standardised 5-year time point, model-predicted IOP was 13.55 mmHg (95% CI: 12.71–14.40) in controls and 14.39 mmHg (95% CI: 13.56–15.21) in the POAG group, representing reductions of 1.25 mmHg and 1.10 mmHg from the respective model-estimated baselines, closely matching the observed mean reductions of 1.20 and 1.13 mmHg, with overlapping confidence intervals and no statistically significant between-group differences.

**Figure 2:**
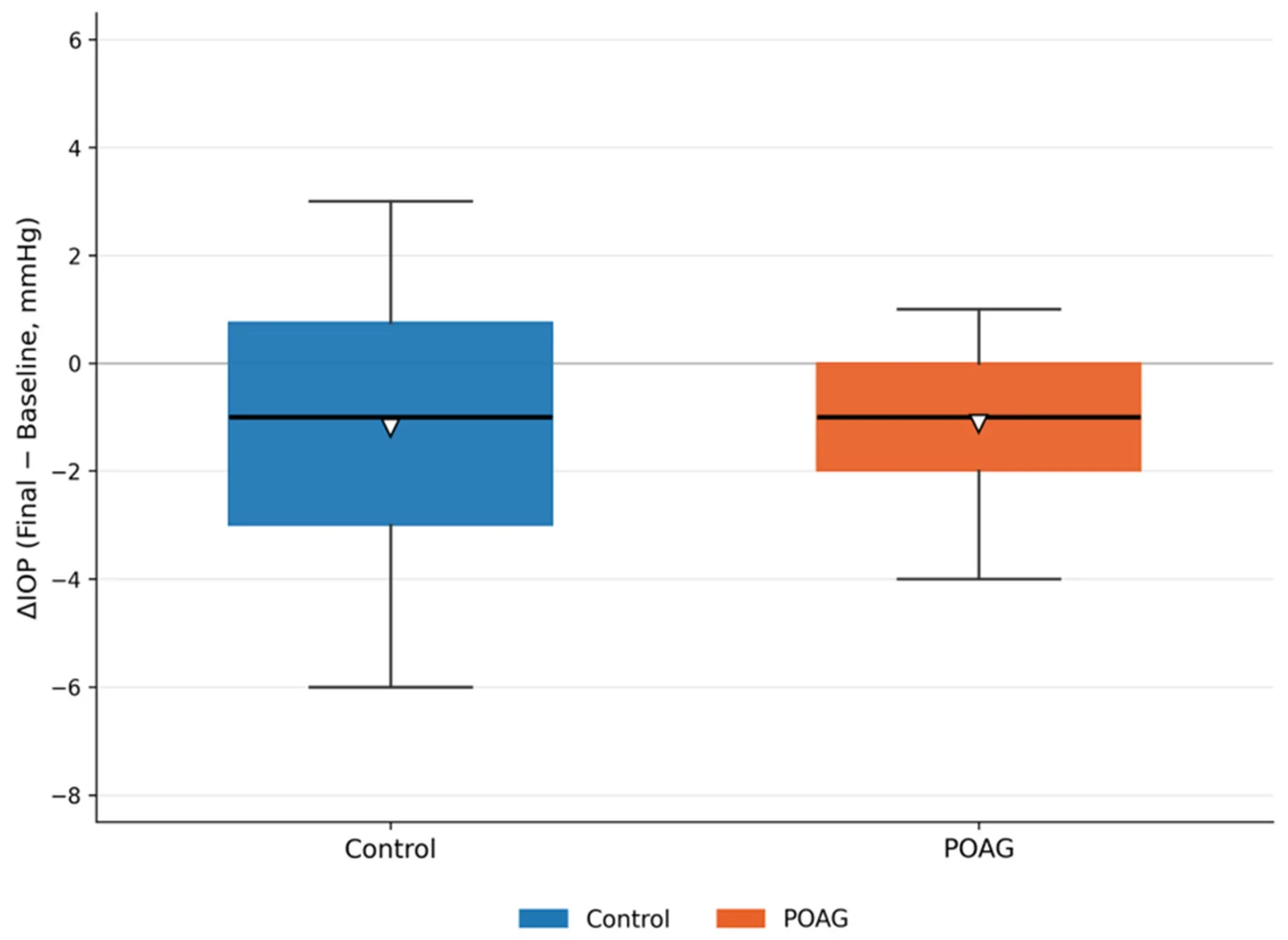
Distribution of intraocular pressure change (ΔIOP, final minus baseline) in control and primary open-angle glaucoma (POAG) eyes. Boxes span the interquartile range (25th–75th percentile); the solid horizontal line within each box denotes the median and the superimposed marker denotes the mean ΔIOP. Whiskers extend to the most extreme values within 1.5 times the interquartile range. Negative values indicate a reduction in intraocular pressure relative to baseline. All 61 eyes (30 control, 31 POAG) are included.

When changes in IOP relative to baseline were analysed, the mean change from baseline at the final follow-up visit was −1.20 mmHg (−8.1%; 95% CI: −2.07 to −0.33) in the control group and −1.13 mmHg (−7.3%; 95% CI: −1.86 to −0.40) in the POAG group; the between-group difference in change was −0.07 mmHg (95% CI: −1.16 to 1.02). No statistically significant differences in IOP reduction between the control and POAG groups were present at any postoperative time point (all *p* > 0.05). The distribution of individual IOP changes from baseline to the final follow-up visit is shown in Figure 2. Substantial inter-individual variability was observed in both groups, with considerable overlap in response ranges, indicating that individual postoperative IOP behaviour varied widely irrespective of glaucoma status.

In the primary open-angle glaucoma group, the mean number of antiglaucoma medications was 1.48 ± 0.68 preoperatively and 1.55 ± 0.68 at the final follow-up visit. Twenty-nine of 31 patients (93.5%) had no change in the number of agents, two required one additional agent, and none reduced therapy (Supplementary Table S1). Because only two paired differences were non-zero, formal testing is of limited value; the approximate Wilcoxon signed-rank test did not detect a statistically significant change (*p* = 0.157), and an exact sign test on the two non-zero differences likewise did not (*p* = 0.50). These data indicate that medication burden changed little over long-term follow-up, but they do not establish equivalence.

In multivariable linear regression analysis evaluating predictors of long-term IOP change (defined as final follow-up IOP minus preoperative IOP), higher preoperative IOP was independently associated with greater numerical long-term IOP reduction (*β* = −0.44 per mmHg; 95% CI: −0.61 to −0.27; *p* < 0.001). Other evaluated demographic and baseline ocular parameters, including age, anterior chamber depth, axial length, lens thickness, keratometry values, corneal astigmatism, intraocular lens power, and preoperative visual acuity, were not significantly associated with long-term IOP change (all *p* > 0.05). The two prespecified postoperative parameters were additionally evaluated and were likewise unrelated to long-term IOP change: anterior chamber deepening at one month (mean 0.96 ± 0.23 mm; *β* = −0.40, *p* = 0.74) and best-corrected visual acuity at one month (*β* = 2.21, *p* = 0.48). Notably, lens thickness was not significantly associated with long-term IOP change (*β* = −0.10 per mm; 95% CI: −1.58 to 1.39; *p* = 0.895), and its inclusion did not alter the model fit (*R^2^* = 0.40). Full regression output for the baseline multivariable model is presented in Table 4. Three additional robustness checks were performed. Adding study group and duration of final follow-up to the model left the preoperative-IOP coefficient essentially unchanged (*β* = −0.47 per mmHg; 95% CI: −0.65 to −0.29; *p* < 0.001), and neither group (*β* = 0.54; 95% CI: −0.54 to 1.63; *p* = 0.318) nor follow-up duration (*β* = −0.03 per month; 95% CI: −0.10 to 0.05; *p* = 0.456) was significantly associated with long-term IOP change. Excluding the two POAG patients whose medication count increased also left the coefficient unchanged (*β* = −0.44; 95% CI: −0.62 to −0.27; *p* < 0.001; *n* = 59). Variance inflation factors were below 3.5 for all predictors (highest: axial length 3.35 and IOL power 2.85), indicating no serious collinearity. The model accounted for 40% of the variance in long-term IOP change (*R^2^* = 0.40; adjusted *R*^2^ = 0.29; overall model *p* = 0.001). Because long-term IOP change was modelled against its own preoperative baseline, the same model was additionally expressed in the analysis-of-covariance parameterisation, with final follow-up IOP as the dependent variable and preoperative IOP entered as a covariate alongside the same baseline parameters (Supplementary Table S2). In this specification, preoperative IOP was strongly associated with final IOP (*β* = 0.56 per mmHg; 95% CI: 0.39 to 0.73; *p* < 0.001); final IOP increased by approximately 0.56 mmHg for every 1 mmHg higher preoperative IOP. Because this coefficient lies below unity, eyes with higher baseline pressure showed a greater numerical reduction from baseline, although they did not necessarily reach lower absolute postoperative pressures; the corresponding change-score slope (*β* − 1 = −0.44) is identical to that of the primary model. This is an algebraically equivalent re-parameterisation of the primary model rather than an independent sensitivity analysis: with the same covariates, all non-baseline coefficients, standard errors and *p*-values are identical, and the baseline coefficient differs by exactly 1.0. It is therefore reported as an alternative and more readily interpretable parameterisation of the same fitted relationship (*R^2^* = 0.48) and does not by itself remove regression-to-the-mean concerns, although a residual contribution from regression to the mean cannot be entirely excluded.

## 4. Discussion

Phacoemulsification produced a modest, sustained drop in IOP in both groups, and the temporal trajectory did not differ meaningfully between eyes with and without medically controlled POAG. Perhaps equally relevant for clinical practice: glaucoma patients reached the end of follow-up with essentially the same topical medication burden with which they started. Although treatment regimens changed in four patients, only two experienced an increase in the number of active agents. Whatever pressure benefit cataract surgery confers in this population, it was accompanied by little overall change in medication burden during follow-up.

The IOP course we observed, involving an early postoperative fall, partial recovery over the following months, and eventual stabilisation below the preoperative level, matches what has been repeatedly described after uncomplicated phacoemulsification [7,8,9,21,22,23,29]. Transient early spikes are well documented, particularly in eyes with glaucoma or elevated baseline pressure [7,11,12,13,14], but in the absence of complications, the longer-term trend is toward a modest reduction. The lack of a significant group–time interaction beyond the first postoperative week indicates that POAG status did not materially alter this pattern. The pressure curves in glaucoma and control eyes ran essentially in parallel across the intermediate and long-term follow-up, aside from a non-significant trend toward a blunted early reduction in glaucomatous eyes (interaction coefficient +2.70 mmHg/month; *p* = 0.061).

The absolute pressure reductions we measured were small, and the scatter around the group means was wide: some eyes showed no change and a few showed an increase, although the overall effect of time on IOP was statistically significant in the mixed-effects model (*p* < 0.001). This degree of inter-individual variability has been consistently noted in the literature on open-angle glaucoma [21,22,23,26,27,28]. A recent systematic review and meta-analysis of randomised trial arms reported a mean IOP reduction of 3.77 mmHg twelve months after phacoemulsification in open-angle glaucoma, exfoliative glaucoma and ocular hypertension, but found that the effect was substantially larger when a medication washout was used (5.25 mmHg) than when it was not (1.81 mmHg after exclusion of an outlier study), indicating that concurrent topical therapy masks part of the pressure-lowering effect [35]. This is directly relevant to our cohort, in which all glaucomatous eyes remained on medication throughout. It is part of why the procedure cannot be relied upon as primary glaucoma surgery for this subtype. A Bayesian pooled analysis found that phacoemulsification in POAG was associated with only a small IOP reduction, with only a 0.1% to 3% probability of achieving a ≥5 mmHg decrease [27]. In their long-term cohort study of normotensive and ocular hypertensive eyes, Poley et al. reported final mean reductions of 4.8 mmHg, 2.5 mmHg, and 1.6 mmHg for baseline 20–22, 18–19, and 15–17 mmHg groups, respectively, while the 9–14 mmHg group increased by 0.2 mmHg [28]. Our mean reductions of 1.20 mmHg in control eyes and 1.13 mmHg in POAG eyes are of the same modest order, consistent with the low mean preoperative pressures in our cohort.

The contrast with angle-closure and pseudoexfoliative glaucoma is worth stating plainly because it matters for surgical planning. In those conditions, phacoemulsification regularly produces IOP reductions that are clinically substantial and durable [15,16,17,18,19,20]. In angle-closure glaucoma, removing a thickened lens directly eliminates the anatomical crowding driving pressure elevation [15,16,17], while in pseudoexfoliation, clearing of lens epithelium and exfoliative material from the trabecular meshwork is thought to restore outflow, although the supporting series combined cataract surgery with trabecular aspiration or washout of exfoliative material rather than phacoemulsification alone [19,20]. POAG works differently: the outflow obstruction sits in the trabecular meshwork and distal channels and is not meaningfully relieved by lens removal [18,21,22,23,26,27]. Direct comparison of the IOP-lowering effect of phacoemulsification in POAG with that observed in angle-closure glaucoma may therefore be inappropriate, given the fundamentally different pathophysiological mechanisms underlying pressure elevation in these two conditions.

Of the preoperative and early postoperative variables we examined, only higher baseline IOP predicted greater long-term pressure reduction. Anterior chamber depth, axial length, lens thickness, keratometry, corneal astigmatism, IOL power, age, and early postoperative anterior chamber deepening and visual acuity were not useful predictors. This agrees with what most other groups examining preoperative predictors have found [18,21,22,23,26,28]. The baseline IOP relationship may partially reflect regression to the mean; the analysis-of-covariance parameterisation (Supplementary Table S2) expresses the same fitted relationship with final IOP as the outcome and does not, on its own, exclude that possibility. Still, there may also be a biological contribution: eyes with higher starting pressures may have more trabecular reserve available to respond to whatever outflow enhancement phacoemulsification produces through stretching of the trabecular meshwork and Schlemm’s canal or changes in zonular and ciliary body tension [30,31]. The practical implication, subject to the exploratory nature of our regression model, is that most commonly used anterior segment biometry parameters do not reliably separate patients who will respond well from those who will not.

For the ophthalmologist managing a patient with early or moderate medically controlled POAG and a visually significant cataract, these data are reassuring. Operating for visual reasons will, on average, provide a modest additional pressure benefit that was sustained over follow-up ranging from 44 to 77 months, with little change in the number of topical agents over the same period. This is worth knowing when counselling older patients, in whom cataract and glaucoma frequently coincide and in whom the outcomes of cataract surgery are well described [2,3,4]. The data do not support using phacoemulsification as a substitute for glaucoma surgery when the target pressure is not being met. In eyes with progressive disease at low IOP, or where a substantial further reduction is needed, additional glaucoma intervention may be required; randomised evidence supports combining phacoemulsification with a trabecular micro-bypass stent for greater pressure reduction than phacoemulsification alone [24,25], and the American Academy of Ophthalmology review reached a similar conclusion regarding the limits of cataract surgery alone [12]. The modest average effects we observed are consistent with that conclusion.

Several methodological features of this study deserve mention. All operations were performed by a single surgeon using the same incision geometry, technique, and postoperative regimen, which removes between-surgeon and between-protocol variability that complicates interpretation in multicentre series. The analytical approach—a linear mixed-effects model with a piecewise time structure—was chosen specifically because the follow-up visits were irregularly spaced and the timing of the final visit differed between patients; simpler approaches applied to this kind of data tend to ignore available information or to treat visits occurring at substantially different times as equivalent, as in several earlier series that relied on time-point-wise comparisons [21,22,23,26]. Including a non-glaucomatous control arm that underwent identical surgery also allowed us to describe the trajectory difference directly rather than infer it from population comparisons.

There are notable limitations. Sixty-one patients from one centre is a modest sample, and 39 of the originally enrolled 100 did not return for long-term follow-up. The attrition analysis gave us confidence that those who returned were not systematically different in IOP from those who did not. Still, a small residual selection bias cannot be ruled out, particularly given the difference in preoperative anterior chamber depth between the control subgroup and the rest of the cohort. The design also combined prospective and retrospective components, each with its own ethics approval: the perioperative and early postoperative data were collected prospectively under a standardised protocol as part of a doctoral research project, while the long-term IOP values were ascertained retrospectively from routine clinical records under a separate, later approval. Measurement conditions were nonetheless kept consistent: all readings were obtained by Goldmann applanation tonometry on the same tonometer and at the same time of day (±2 h), which limits diurnal and instrument-related variability. Residual limitations of the retrospective component remain, however—the timing of the long-term visit varied between patients and the examiner was not necessarily the same at every visit—so a degree of measurement variability and ascertainment bias cannot be entirely excluded.

Our glaucoma cohort was well-controlled and early-to-moderate by design; we cannot say how the findings would translate to patients with advanced disease, uncontrolled pressure, or other glaucoma subtypes. We tracked medication as a count of topical agents—a reasonable proxy but one that misses adherence, dosing changes within the same drug class, and concentration differences. Because long-term IOP change was defined as final follow-up minus preoperative IOP while preoperative IOP was simultaneously entered as a predictor, the strong association between higher baseline IOP and greater reduction partly reflects mathematical coupling and regression to the mean rather than a purely biological effect; we therefore also report the analysis-of-covariance parameterisation, with final IOP as the outcome and preoperative IOP as a covariate (Supplementary Table S2; β = 0.56; 95% CI: 0.39 to 0.73; *p* < 0.001). Because that model is algebraically equivalent to the change-score model, it clarifies interpretation but does not independently exclude regression to the mean; only repeated baseline measurements or an external reliability estimate could do so. Entering nine predictors for 61 patients also yields fewer than ten observations per variable, below the conventional threshold for stable estimation, so the multivariable regression should be regarded as exploratory and potentially subject to overfitting. Biometry was performed using contact ultrasound A-scan, which can slightly compress the anterior chamber and marginally underestimate anterior chamber depth and axial length relative to optical or immersion techniques; because the same method was applied uniformly to all eyes, this is unlikely to have biased the between-group comparisons or the predictor analyses. Finally, we measured IOP, not visual field or optic nerve structure; whether the pressure changes we observed have downstream consequences for glaucoma progression requires longer follow-up and structural data that were not part of this study.

What would move this field forward is a multicentre, fully prospective study with prespecified long-term follow-up, standardised anterior segment imaging, and concurrent visual field assessment—large enough to stratify by baseline IOP and disease severity and to detect clinically meaningful differences between phacoemulsification alone and combined phacoemulsification with minimally invasive glaucoma surgery (MIGS) [12,24,25]. Modelling IOP and structural progression jointly would also help translate pressure trajectories of the kind we report into estimates of glaucoma control that are directly relevant to clinical decision-making.

## 5. Conclusions

Uncomplicated phacoemulsification in eyes with and without medically controlled primary open-angle glaucoma resulted in a modest but sustained reduction in IOP over long-term follow-up. No statistically significant difference in the magnitude or temporal pattern of IOP change was detected between glaucomatous and non-glaucomatous eyes, although the confidence intervals do not establish equivalence. In the POAG group, mean IOP remained below the preoperative group mean at every scheduled postoperative assessment, and 29 of 31 patients required no change in the number of topical agents. While cataract surgery alone provides limited pressure-lowering benefit in medically controlled POAG, the modest reduction it produces is maintained over long-term follow-up without a statistically significant increase in medication burden.

## Figures and Tables

**Table 1:** Baseline demographic and ocular characteristics of the study population.

Variable	Control (n = 30)	POAG (n = 31)	*p*-Value
Age (years)	72.83 ± 6.39	74.55 ± 7.80	0.351
Female sex, n (%)	18 (60.0)	17 (54.8)	0.797
Follow-up (months)	56.8 ± 8.2 (44–77)	58.0 ± 6.7 (48–71)	0.523
Preoperative IOP (mmHg)	14.80 ± 2.50	15.48 ± 3.12	0.348
ACD preoperative (mm)	3.23 ± 0.32	3.02 ± 0.28	0.010
Axial length (mm)	23.38 ± 1.02	23.60 ± 1.48	0.891
Lens thickness (mm)	4.40 ± 0.38	4.57 ± 0.34	0.070
K1 (D)	42.65 ± 1.59	42.53 ± 1.42	0.767
K2 (D)	42.78 ± 1.55	42.76 ± 1.23	0.959
Corneal astigmatism (D)	0.38 ± 0.40	0.66 ± 0.75	0.151
IOL power (D)	21.30 ± 2.08	21.43 ± 2.34	0.375
BCVA preoperative (decimal)	0.19 ± 0.17	0.21 ± 0.21	0.959

Values are mean ± standard deviation unless otherwise stated. Continuous variables were compared using Welch’s *t*-test when both groups passed the Shapiro–Wilk normality test and the Mann–Whitney U test otherwise; sex was compared using Fisher’s exact test. POAG = primary open-angle glaucoma; IOP = intraocular pressure; ACD = anterior chamber depth; BCVA = best-corrected visual acuity; IOL = intraocular lens.

**Table 2:** Fixed effects from the linear mixed-effects model assessing longitudinal IOP (mmHg) changes.

Effect	Estimate	SE	z-Value	*p*-Value	95% CI
Intercept	14.80	0.42	34.95	<0.001	13.97 to 15.63
Early postoperative slope	−7.82	1.03	−7.62	<0.001	−9.83 to −5.81
Intermediate slope	0.09	0.05	1.89	0.059	−0.003 to 0.179
Long-term slope	0.004	0.005	0.69	0.488	−0.01 to 0.01
POAG (group effect)	0.68	0.59	1.15	0.250	−0.48 to 1.85
Early slope × POAG	2.70	1.44	1.87	0.061	−0.13 to 5.52
Intermediate slope × POAG	−0.04	0.07	−0.61	0.545	−0.17 to 0.09
Long-term slope × POAG	−0.006	0.008	−0.73	0.466	−0.02 to 0.01

A random intercept for the subject was included to account for repeated measurements. The intercept represents the estimated baseline IOP for the reference group (patients without primary open-angle glaucoma). Estimates are expressed in mmHg; slope coefficients represent the change in IOP (mmHg) per month within each predefined time segment. The model was fit using restricted maximum likelihood to 366 observations from 61 subjects. The random-intercept variance was 4.08 mmHg^2^ and the residual variance 1.30 mmHg^2^, corresponding to an intraclass correlation of 0.76 (approximately 76% of the total variance reflecting stable between-eye differences).

**Table 3:** Model-based predicted IOP values at selected postoperative time points. Values are fixed-effect predicted group means from the piecewise linear mixed-effects model with 95% confidence intervals for the estimated mean (not prediction intervals for an individual eye).

Time Point	Control (mmHg)	POAG (mmHg)
Baseline	14.80 (13.97–15.63)	15.48 (14.67–16.30)
1 week	12.84 (12.06–13.62)	14.20 (13.43–14.97)
1 month	12.91 (12.15–13.67)	14.24 (13.49–14.99)
3 months	13.09 (12.33–13.84)	14.34 (13.60–15.07)
6 months	13.35 (12.54–14.16)	14.48 (13.68–15.28)
60 months	13.55 (12.71–14.40)	14.39 (13.56–15.21)

Values are model-predicted means with 95% confidence intervals. The values at 60 months represent model-derived IOP estimates at a standardised 5-year time point within the observed follow-up range.

**Table 4:** Multivariable linear regression analysis of predictors of long-term IOP change.

Predictor	*β*	SE	95% CI	*p*-Value
Preoperative IOP (per mmHg)	−0.44	0.08	−0.61 to −0.27	<0.001
Age (per year)	−0.03	0.04	−0.10 to 0.05	0.465
Anterior chamber depth, preop. (per mm)	0.25	0.83	−1.41 to 1.91	0.765
Axial length (per mm)	0.23	0.34	−0.45 to 0.90	0.505
Lens thickness (per mm)	−0.10	0.74	−1.58 to 1.39	0.895
Mean keratometry (per D)	0.31	0.21	−0.12 to 0.74	0.151
Corneal astigmatism (per D)	0.34	0.42	−0.50 to 1.18	0.425
IOL power (per D)	0.11	0.18	−0.25 to 0.47	0.542
Preoperative BCVA (per decimal unit)	−0.91	1.62	−4.17 to 2.35	0.578
Intercept	−13.78	18.15	−50.22 to 22.66	0.451

The dependent variable was long-term IOP change, defined as the difference between final follow-up and preoperative IOP; negative coefficients indicate a greater long-term reduction in IOP. All nine candidate predictors were entered simultaneously into a single model (n = 61). Model R^2^ = 0.40; adjusted R^2^ = 0.29; overall model *p* = 0.001 (F-test). Keratometry was entered as mean keratometry. β = unstandardised regression coefficient; SE = standard error; CI = confidence interval; IOL = intraocular lens; BCVA = best-corrected visual acuity; D = dioptres.

## Data Availability

The data presented in this study are available on request from the corresponding author. The data are not publicly available due to privacy and ethical restrictions relating to patient data.

## Supplementary Materials

The following supporting information can be downloaded at: https://www.mdpi.com/article/10.3390/medicina62081525/s1, Supplementary Table S1: Changes in antiglaucoma medication burden in the POAG group; Supplementary Table S2: Alternative parameterisation of the multivariable model using final follow-up IOP as the dependent variable.
