# Supplementary material for: Long-Term Intraocular Pressure Changes Following Phacoemulsification in Eyes with and Without Primary Open-Angle Glaucoma
Source: Medicina (Kaunas). 2026 Aug 8;62(8):1525. doi: 10.3390/medicina62081525 (PMC13514959; doi:10.3390/medicina62081525)
Supplement: Supplementary file 1 [file medicina-62-01525-s001.zip › medicina-4442329-supplementary.pdf]

Supplementary Table S1. Changes in antiglaucoma medication burden in the POAG group

Patient No.	Antiglaucoma therapy — preoperative	No. of medications preoperative	Antiglaucoma therapy — final follow-up	No. of medications at final follow-up	Change (Δ)	Follow-up (months)
1	Latanoprost	1	Latanoprost	1	0	58
2	Travoprost	1	Travoprost	1	0	67
3	Latanoprost + timolol/dorzolamide	3	Latanoprost + timolol/dorzolamide	3	0	64
4	Latanoprost	1	Latanoprost	1	0	55
5	Timolol/dorzolamide	2	Timolol/dorzolamide	2	0	56
6	Travoprost	1	Travoprost	1	0	53
7	Latanoprost	1	Latanoprost	1	0	52
8	Latanoprost	1	Latanoprost	1	0	48
9	Latanoprost + timolol	2	Latanoprost + timolol	2	0	60
10	Latanoprost + timolol	2	Latanoprost + timolol	2	0	48
11	Timolol	1	Latanoprost	1	0	55
12	Latanoprost + timolol/dorzolamide	3	Latanoprost + timolol/dorzolamide	3	0	54
13	Latanoprost + timolol/dorzolamide	3	Latanoprost + timolol/dorzolamide	3	0	61
14	Latanoprost	1	Latanoprost	1	0	49
15	Timolol/dorzolamide	2	Timolol/dorzolamide	2	0	63
19	Timolol/dorzolamide	2	Timolol/dorzolamide	2	0	54
21	Latanoprost	1	Latanoprost	1	0	66

Patient No.	Antiglaucoma therapy — preoperative	No. of medications preoperative	Antiglaucoma therapy — final follow-up	No. of medications at final follow-up	Change (Δ)	Follow-up (months)
25	Latanoprost	1	Latanoprost	1	0	59
31	Dorzolamide + latanoprost	2	Dorzolamide + latanoprost	2	0	55
34	Timolol/dorzolamide	2	Timolol/dorzolamide	2	0	48
35	Timolol/dorzolamide	2	Timolol/dorzolamide	2	0	69
36	Latanoprost	1	Latanoprost	1	0	60
39	Travoprost	1	Travoprost	1	0	48
40	Latanoprost	1	Latanoprost	1	0	60
41	Latanoprost	1	*Timolol + bimatoprost*	**2**	**+1**	70
42	Tafluprost	1	Latanoprost	1	0	58
43	Tafluprost	1	Tafluprost	1	0	66
44	Timolol + bimatoprost	2	Timolol + bimatoprost	2	0	58
45	Timolol	1	*Timolol/dorzolamide*	**2**	**+1**	54
47	Travoprost	1	Travoprost	1	0	71
48	Travoprost	1	Travoprost	1	0	60
**Mean ± SD**	—	**1.48 ± 0.68**	—	**1.55 ± 0.68**	**+0.06**	**58.0 ± 6.7**

No. = number; Δ = change (final follow-up minus preoperative); POAG = primary open-angle glaucoma. Bold italic entries indicate patients with an increase in medication burden. Of 31 patients, 29 showed no change in the number of antiglaucoma medications. Two patients (Nos. 41 and 45) required an additional agent. No patient reduced their medication burden. The mean change (+0.06) corresponds to two single-step increases across 31 patients (2/31 = 0.065); the

small discrepancy from the difference of the rounded group means (1.55 − 1.48) is due to rounding. Because only two paired differences were non-zero, the Wilcoxon signed-rank result is based on the normal approximation and should be interpreted cautiously ($p = 0.157$); an exact two-sided sign test of the two non-zero differences gave $p = 0.50$. The descriptive counts are therefore the primary result. Patient numbers correspond to identifiers in the original 100-patient cohort and are therefore nonconsecutive.

Supplementary Table S2. Alternative parameterisation of the multivariable model using final follow-up IOP as the dependent variable

Predictor	β	SE	95% CI	p-value
Preoperative IOP (per mmHg)	0.56	0.09	0.39 to 0.73	< 0.001
Age (per year)	−0.03	0.04	−0.10 to 0.05	0.465
Anterior chamber depth, preop. (per mm)	0.25	0.83	−1.41 to 1.91	0.765
Axial length (per mm)	0.23	0.34	−0.45 to 0.90	0.505
Lens thickness (per mm)	−0.10	0.74	−1.58 to 1.39	0.895
Mean keratometry (per D)	0.31	0.21	−0.12 to 0.74	0.151
Corneal astigmatism (per D)	0.34	0.42	−0.50 to 1.18	0.425
IOL power (per D)	0.11	0.18	−0.25 to 0.47	0.542
Preoperative BCVA (per decimal unit)	−0.91	1.62	−4.17 to 2.35	0.578
Intercept	−13.78	18.15	−50.22 to 22.66	0.451

Analysis of covariance with final follow-up IOP as the dependent variable and preoperative IOP entered as a covariate alongside the same baseline parameters as in Table 4 (n = 61). A coefficient of 0.56 indicates that final IOP increased by approximately 0.56 mmHg for every 1 mmHg higher preoperative IOP. Because the coefficient lies below unity, eyes with higher baseline pressure showed a greater numerical reduction from baseline, although they did not necessarily reach lower absolute postoperative pressures. This model is an algebraically equivalent re-parameterisation of the change-score model in Table 4; the corresponding change-score coefficient is $\beta - 1 = -0.44$. Model $R^2 = 0.48$; adjusted $R^2 = 0.39$; overall model $p < 0.001$ (F-test). β = unstandardised regression coefficient; SE = standard error; CI = confidence interval; IOP = intraocular pressure; IOL = intraocular lens; BCVA = best-corrected visual acuity; D = dioptres.
